# Supplementary material for: Nitrogen Self-Doping Carbon Derived from Functionalized Poly(Vinylidene Fluoride) (PVDF) for Supercapacitor and Adsorption Application
Source: Micromachines (Basel). 2022 Oct 15;13(10):1747. doi: 10.3390/mi13101747 (PMC9611783; doi:10.3390/mi13101747)
Supplement: Supplementary file 1 [file micromachines-13-01747-s001.zip › micromachines-1952584-supplementary.pdf]

Nitrogen Self-doping Carbon Derived from Functionalized Poly(vinylidene fluoride) (PVDF) for High-Performance Supercapacitor Electrodes

Yantao Zheng[#2], Qifei Liu[#1], Xingyu Guan[1,3], Yuan Liu[1], Shengqiang Nie[1]* and Yi Wang[1]*

[1]*College of Chemistry and Material Engineering, Guiyang University, 550005, Guiyang, P.R. China*

[2] *Xifeng Phosphorite Mine Co., Ltd., Guiyang, 551100, China*

[3]*Saint Petersburg State Technical University, 190013, Saint Petersburg, Russia*

[#]*These two authors contribute equally to this work*

[*]*Corresponding author*

E-mail address: nieshq1987@163.com

Electrochemical measurements

The CHI660E electrochemical workstation was used to conduct electrochemical measurements on carbon compounds in a 6 M KOH solution. The Pt sheet and the Hg/HgO electrode were utilized as the counter and the reference electrodes. The prepared carbon materials (mass ratio 80%), carbon black(mass ratio 10%) and PVDF(mass ratio 10%) was mixed and loaded on a nickel foam(1×1 cm^2) to fabricate the working electrode. The electrochemical impedance spectroscopy (EIS) was performed over a range of 0.01 Hz–100 kHz. The electrolyte is 6M KOH, mass loading of carbon and n-doped carbon is 4 mg respectively. The current collector is Ni foam.

The equations used for the calculation of specific capacitance and for absorption are added in the supporting information.The values of capacitance were deduced from through followed equations.

$$C\ (F/g) = I\times\Delta t/m\Delta V \qquad (1)$$

where I (A), Δt (s), m (g) and ΔV (V) on behalf of the discharge current, time for discharge, mass of the carbon material and voltage window, respectively.

For the supercapacitor device, the energy density E (Wh/kg) and power density P (W/kg) were calculated based on the following equation:

$E = 0.5 \times Cs \times \Delta V^2 /3.6$ (2)

$P = 3600\ E / \Delta t.$ (3)

Where Cs (F g-1), ΔV (V) Δt (s) denote the specific capacitance, the potential change, the discharging time.

Adsorption experiment

Methylene blue (MB) and methyl orange (MO) were used as probe dyes to discover the adsorption capability of NC-800. In brief, 200 mg of NC-800 was added to 200 mL dye solutions (200 mg/L). The dye present in the solution was sampled at regular intervals, centrifuged, and the concentration was determined using a UV–vis spectro-photometer (LabTech, Beijing) at 620 nm and 540 nm, respectively.

We calculated the adsorption amounts and adsorption ratios of MB and MO using the equation described yellow:

Adsorption amounts (mg) = $(C_0V_0 - C_tV_t)$ (1)

Adsorption ratios (mg/g) = $(C_0V_0 - C_tV_t)/m_0$ (2)

Where V_0 and C_0 denote the volume and concentration of the initial MB and MO solution; V_t and C_t were the volume and concentration of the MB and MO solution at regular intervals; m_0 was the initial heteroatom-doped porous carbon mass.

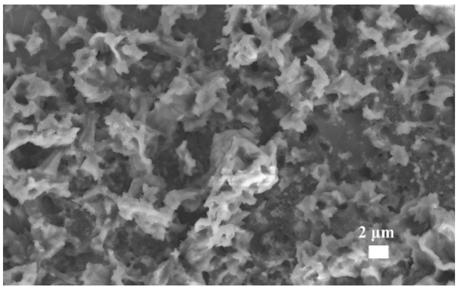

Figure S1 The SEM image of carbon
